# Supplementary material for: Content-rich biological network constructed by mining PubMed abstracts
Source: BMC Bioinformatics. 2004 Oct 8;5:147. doi: 10.1186/1471-2105-5-147 (PMC528731; doi:10.1186/1471-2105-5-147)
Supplement: Additional File 2 — The original results of the above study (non-essential files are deleted to keep the file size under the limit set by BMC bioinformatics). [file 1471-2105-5-147-S2.bz2 › chilibotAdditionalFile2/dip20/579ID9437425E10947/html/CHEY_CHEA.html]

 


 **CHEY** and **CHEA** 
  
Found 134 abstracts in PubMed, retrieved 20.  
 

 What does Google say? 
 PDF only 
| .edu only 

---

**Interactive relationship** (e.g. stimulation, inhibition, etc)

**Neutral relationship**- **CheA**   **CheY**  phosphotransfer generates phospho  **CheY** ,  **CheY**  P.  Ref: 11243808 J Mol Biol, 2001
- Autophosphorylated  **CheA**  passes its phosphoryl group to  **CheY**  very rapidly k cat approximately 750 s 1.  Ref: 11052668 Biochemistry, 2000
- **CheY**  is phosphorylated on Asp57 by the sensor kinase  **CheA**  and dephosphorylated by CheZ.  Ref: 12591865 J Bacteriol, 2003
- Reverse transcription PCR confirmed that the other downstream chemotaxis genes within the same operon cheW, cheX, and  **cheY**  were still expressed in the  **cheA**  mutant strain.  Ref: 12003957 J Bacteriol, 2002
- The protein  **CheY**  shuttles between flagellar motors and methyl accepting chemoreceptor MCP complexes containing the linker CheW and the kinase  **CheA** .  Ref: 11243808 J Mol Biol, 2001
- Here we use fluorescence resonance energy transfer to monitor interactions of  **CheY**  approximately P with its phosphatase, CheZ, that reveal changes in the activity of the receptor kinase,  **CheA** , resulting from the addition of attractants or repellents.  Ref: 11742065 Proc Natl Acad Sci U S A, 2002
- Binding of an attractant to the chemoreceptors inhibits  **CheA** , which results in decreased phosphorylation of the chemotaxis response regulator  **CheY** .  Ref: 12119291 J Biol Chem, 2002

**Non-interactive relationship** (e.g. studied together, co-existance, homology, etc.)

- In bacteria, these receptors include the transmembrane sensor kinases, which control gene expression via their cognate response regulators, and chemoreceptors, which control the direction of flagellar rotation via the  **CheA**  kinase and  **CheY**  response regulator.  Ref: 11994152 Mol Microbiol, 2002
- Although phosphotransfer was slower with CheADeltaP2 k cat K m approximately 1.5 x 10 6 M 1 s 1 than with wild type  **CheA**  k cat K m approximately 10 8 M 1 s 1, it was still orders of magnitude faster than the kinetics of  **CheY**  phosphorylation by phosphoimidazole and other small molecule phosphodonors k cat K m approximately 5 50 M 1 s 1.  Ref: 11052668 Biochemistry, 2000
- The objective of this study was to probe the conformational coupling between the phosphorylation site and the signaling surface of  **CheY**  in the reverse direction by quantifying phosphorylation activity in the presence and absence of peptides of  **CheA** , CheZ, and FliM that specifically interact with  **CheY** .  Ref: 11353835 Proc Natl Acad Sci U S A, 2001
- The third locus encodes two  **CheA** , one CheR, one CheB, one CheW, one  **CheY** , a putative cytoplasmic chemoreceptor TlpT and a protein showing homology to the chromosomal partitioning factor Soj designated Slp.  Ref: 12421313 Mol Microbiol, 2002
- Further insights into the mechanism of function of the response regulator  **CheY**  from crystallographic studies of the  **CheY**   **CheA**  124 257 complex.  Ref: 11134926 Acta Crystallogr D Biol Crystallogr, 2001
- It is strongly suggested that the positive phototactic response was mediated by a phytochrome like photoreceptor and  **CheA**   **CheY**  type signal transduction system.  Ref: 11134414 Plant Cell Physiol, 2000
- New crystallographic structures of the response regulator  **CheY**  in association with  **CheA**  124 257, its binding domain in the kinase  **CheA** , have been determined.  Ref: 11134926 Acta Crystallogr D Biol Crystallogr, 2001
- Past experiments have shown that  **CheA**  and  **CheY**  are required to generate smooth swimming signals in Bacillus subtilis chemotaxis.  Ref: 12094812 Microbiology, 1997
- These results indicate that binding of  **CheY**  to the P2 domain of  **CheA**  indeed contributes to the rapid kinetics of phosphotransfer.  Ref: 11052668 Biochemistry, 2000
- This study, as anticipated from in vivo experiments, demonstrates in vitro that an attractant bound chemoreceptor leads to an increase in  **CheA**  activity, which in turn leads to an increase in the  **CheY**  P pool that ultimately causes a behavioural change in the bacteria.  Ref: 12094812 Microbiology, 1997
- Asparagine has been found to increase the rate of  **CheY**  P formation in the presence of McpB containing membranes,  **CheA** , and an excess of  **CheY** .  Ref: 12094812 Microbiology, 1997
- No phosphoryl group attached to Asp57 of  **CheY**  is visible from the electron density, but the response regulator in the  **CheY**   **CheA**  124 257 complex may have undergone a phosphorylation dephosphorylation process.  Ref: 11134926 Acta Crystallogr D Biol Crystallogr, 2001
- While searching for chemotaxis genes in M. xanthus, we identified a third chemotaxis like gene cluster, the che3 cluster, encoding homologs to two methyl accepting chemotaxis proteins MCPs, a CheW, a hybrid  **CheA** , a CheB, a CheR, but no  **CheY** .  Ref: 12566562 Proc Natl Acad Sci U S A, 2003
- Deletion of either the complete chemosensory operons 1 and 2 plus the response regulator genes  **cheY**  4 and  **cheY**  5 or  **cheA**  2 alone led to the loss of all aerotactic responses, although the cells still swam normally.  Ref: 12270816 J Bacteriol, 2002
- We used rapid quench and stopped flow fluorescence experiments to monitor phosphotransfer to  **CheY**  from phosphorylated wild type  **CheA**  and from phosphorylated CheADeltaP2.  Ref: 11052668 Biochemistry, 2000
- **CheA**  2 phosphorylated all of the CheYs and both CheBs, whilst  **CheA**  1 did not phosphorylate either CheB and phosphorylated only the response regulators encoded within its own genetic locus  **CheY**  1,  **CheY**  2, and  **CheY**  5 and  **CheY**  3.  Ref: 12421557 J Mol Biol, 2002
- The purple photosynthetic bacterium Rhodobacter sphaeroides has three loci encoding multiple homologues of the bacterial chemosensory proteins 13 putative chemoreceptors, four CheW, four  **CheA** , six  **CheY** , two CheB and three CheR.  Ref: 12421313 Mol Microbiol, 2002
- Rapid phosphotransfer to  **CheY**  from a  **CheA**  protein lacking the  **CheY**  binding domain.  Ref: 11052668 Biochemistry, 2000
- The genome sequence of Campylobacter jejuni NCTC 11168 reveals the presence of orthologues of the chemotaxis genes  **cheA** , cheW, cheV,  **cheY** , cheR and cheB, ten chemoreceptor genes and two aerotaxis genes.  Ref: 11912013 Trends Microbiol, 2002
- Together, these results suggest that the binding of  **CheY**  to  **CheA**  124 257 generates a geometry of the active site that favours phosphorylation.  Ref: 11134926 Acta Crystallogr D Biol Crystallogr, 2001
- To explore the contribution of P2 to the  **CheA**  >  **CheY**  phosphotransfer reaction in the Escherichia coli chemotaxis system, we examined the transfer kinetics of a mutant  **CheA**  protein CheADeltaP2 in which the 98 amino acid P2 domain had been replaced with an 11 amino acid linker.  Ref: 11052668 Biochemistry, 2000
